# Environmental Hazards of Boron and Vanadium Nanoparticles in the Terrestrial Ecosystem—A Case Study with *Enchytraeus crypticus*

**DOI:** 10.3390/nano11081937

**Published:** 2021-07-28

**Authors:** Angela Barreto, Joana Santos, Mónica J. B. Amorim, Vera L. Maria

**Affiliations:** Department of Biology & CESAM, University of Aveiro, 3810-193 Aveiro, Portugal; abarreto@ua.pt (A.B.); joanasilvasantos@ua.pt (J.S.); mjamorim@ua.pt (M.J.B.A.)

**Keywords:** nanomaterials, longer-term exposure, soil species, standard test extension, mortality, reproductive output, avoidance behavior

## Abstract

From the start of the 21st century, nanoecotoxicological research has been growing in fast steps due to the need to evaluate the safety of the increasing use of engineered nanomaterials. Boron (B) and vanadium (V) nanoparticles (NPs) generated by anthropogenic activities are subsequently released in the environment; therefore, organisms can be continuously exposed to these NPs for short or long periods. However, the short and long-term effects of BNPs and VNPs on soil organisms are unknown. This work aimed to recognize and describe their potential toxicological effects on the model species *Enchytraeus crypticus*, assessing survival and reproduction, through a longer-term exposure (56 days (d)—OECD test extension of 28 d), and avoidance behavior, through a short-term exposure (48 hours (h)). After 28 d, BNPs did not induce a significant effect on *E. crypticus* survival, whereas they decreased the organisms’ reproduction at 500 mg/kg. From 10 to 500 mg/kg, VNPs decreased the *E. crypticus* survival and/or reproduction. After 56 d, 100 to 500 mg/kg BNPs and 50 to 500 mg/kg VNPs, decreased the reproduction output of *E. crypticus*. The estimated Effect Concentrations (EC_x_) based on reproduction, for BNPs, were lower at 56 d compared with 28 d; for VNPs, an opposite pattern was found: EC_x_ 28 d < EC_x_ 56 d. BNPs did not induce an avoidance behavior, but organisms avoided the soil contaminated with 10 mg VNPs/kg. The tested NPs showed different *E. crypticus* apical effects at 28 d from the ones detected at 56 d, dependent on the type of NPs (B vs. V). In general, VNPs showed to be more toxic than BNPs. However, the effects of VNPs were alleviated during the time of exposure, contrarily to BNPs (which became more toxic with extended duration). The present study adds important information about NPs toxicity with ecological significance (at the population level). Including long-term effects, the obtained results contributes to the improvement of NPs risk assessment.

## 1. Introduction

Engineered nanoparticles (NPs) are the building blocks of engineered nanomaterials (NMs), being smaller than 100 nm in at least one dimension [1]. Due to their unique physicochemical properties, NPs-based products are widely used in many fields. Thus, extensive production and use of NMs ultimately result in their massive release into the environment [2]. Concerning the terrestrial environment, NPs enter mostly via sewage sludge [3]. Nevertheless, the NPs ecotoxicity evaluation is a challenge due to the limited information related to their fate, potential interactions, and behavior in environmental complex matrices (such as soil) [3,4,5]. Once released into the environment, NPs undergo several transformation processes that may change their physicochemical characteristics and consequently their fate, bioavailability, and toxicity to the organisms [6,7].

Boron NPs (BNPs) with distinct functional and structural properties are increasingly employed in a variety of areas such as nuclear technology, electronics, ceramics, healthcare, cosmetics industry, and medical research (in specific, in neutron capture therapy for cancer treatment) [8,9]. Vanadium NPs (VNPs) are applied in diverse electronic devices; specifically, they can be applied in catalysis, electrochromic and optical switching devices, electrochemical capacitors, and windows for solar cells [10,11]. Both NPs, BNPs and VNPs, may be discharged, released and, consequently, accumulated in the environment during synthesis, manufacturing or use of NPs-containing products, being highly relevant to assess their potential toxicity. Lethal effects were observed in honeybees (*Apis mellifera*) after 96 hours (h) BNPs exposure, with a estimated 50% of lethal concentration (LC_50_) of 0.360 mg/L [12]. BNPs, after 24 h exposure, induced 100% of *Daphnia magna* mortality for concentrations above 80 mg/L [13]. For VNPs, reactive oxygen species (ROS) generation, mitochondrial damage and apoptosis were observed in human lung cells [14,15]. Wörle-Knirsch et al. (2007) [16] also reported cell viability reduction and lipid peroxidation. However, no ecotoxicity study with soil organisms was found for these NPs. Concerning non-nano forms, the B element occurs naturally in the soil, being considered an essential micronutrient to plants as well as being nutritionally important for animals. However, B can also be toxic to cells at high concentrations [17,18], though the mechanisms involved in this toxicity are not yet very well understood [17]. The V element occurs as a natural component of the earth crust (in various minerals, coal, and crude oil), but high doses of V can be toxic [19]. Increased generation of ROS and oxidative stress play a predominant role in V-induced cytotoxicity [19].

The avoidance response is considered an extremely relevant ecological endpoint, because if the organisms avoid a contaminated soil, the services provided by them will be compromised and the habitat function declines, negatively affecting the soil ecosystem [20,21,22,23]. On the other hand, if the organisms are not able to avoid the contaminated soil, the hazard on the organisms may be much higher [23,24,25]. Studies were performed assessing the avoidance behavior of soil organisms (e.g., *Eisenia fetida*, *Enchytraeus crypticus*, *Porcellionides pruinosus*, *Porcellio scaber* and *Tenebrio molitor*) when exposed to NPs-contaminated soil [20,22,23,26,27]. However, there are no studies assessing the avoidance behavior of soil organisms exposed to BNPs or VNPs. Organisms can be constantly exposed to NPs for long periods. Thus, performing longer-term exposure studies with NPs is one of the key recommendations in order to guarantee sustainable environmental development [2]. Concerning terrestrial organisms, metal NPs, such as copper oxide and silver, showed mechanisms of toxicity in longer-term exposures that were not predictable based on short-term studies [28,29]. Some longer-term studies assessing the toxicity of different NPs to soil organisms are available (e.g., *E. crypticus* [28,29,30,31,32,33,34,35,36,37] (Table 1) and *Folsomia candida* [38]).

This investigation aimed to discover and elucidate the effects of BNPs and VNPs to the model species *E. crypticus*, assessing survival and reproduction, through 56 days (d) of exposure, and the avoidance behavior, through 48 h of exposure. The current terrestrial toxicity tests are performed based on guidelines standardized by OECD and ISO, usually using a fraction of the life cycle of the test species (e.g., standard Enchytraeid Reproduction Test (ERT)—21 d) [29]. The survival and reproduction were determined after 28 d, starting with juveniles 17–19 d old instead of adults with a well-developed clitellum, as indicated by the standard OECD guideline [39]. An additional 28 d exposure period was tested (resulting in a total of 56 d of exposure) to assess longer-term effects in the reproductive output of the population.

## 2. Material and Methods

### 2.1. Test Organism

*Enchytraeus crypticus* (Enchytraeidae, Oligochaeta), Westheide & Graefe, 1992, was used. The cultures were kept in agar, consisting of Bacti-Agar medium (Agar No. 1, Lab M Limited, Lancashire, UK)) and a mixture of four different salt solutions at final concentrations of 2 mM CaCl_2_·2H_2_O, 1 mM MgSO_4_, 0.08 mM KCl, and 0.75 mM NaHCO_3_, at a temperature of 20 °C with a 16 h:8 h light:dark photoperiod. Cultures were fed on ground-autoclaved oats twice per week. For the extension of the Enchytraeid Reproduction Test (ERT extension), synchronized cultures of *E. crypticus* were prepared by transferring adults with well-developed clitellum into fresh agar plates to lay cocoons. The number of adults to transfer should be two and a half of the number of cocoons required. After 2 d, cocoons were transferred to fresh agar plates. Juveniles with 17–19 d were used. According to Directive 2010/63/EU of the European Parliament and of the Council of 22/9/2010, invertebrates, like *E. crypticus*, are permitted biological models for scientific experimentation and are free of Ethical Statement.

### 2.2. Test Materials and Characterization

Commercial BNPs (Stock No: NS6130-12-001263, CAS: 7440-42-8) and VNPs (Stock No: NS6130-12-001065, CAS: 7440-62-2) dispersions (2% in Triton X-100 and water) were purchased by Nanoshel UK Limited (Cheshire, UK) and were both labelled with an average particle size (APS) between 80 and 100 nm and a purity of 99.9%. NPs dispersions diluted in ultrapure water were characterized by hydrodynamic size, assessed by dynamic light scattering (DLS; Zetasizer Nano ZS, Malvern, UK), and by zeta potential, and evaluated by electrophoretic light scattering (ZP; Zetasizer Nano ZS, Malvern, UK). The Zetasizer Nano ZS (Malvern, UK) also allowed us to obtain the polydispersity index (PDI) of the NPs dispersions.

### 2.3. Test Soil and Spiking Procedures

The natural standard LUFA 2.2 soil (Speyer, Germany) was used for the tests and had the following main characteristics: pH (0.01 M CaCl_2_) = 5.8, organic carbon = 1.71%, cation exchange capacity = 9.2 meq/100 g, maximum water-holding capacity (WHC) = 44.8%, and grain size distribution of 7.2% clay, 8% silt, and 77.5% sand.

The soil was dried (48 h; 60 °C) before use. The control soil was prepared by adding deionized water to adjust to the adequate moisture content (50% of the WHC maximum). Due to the presence of Triton X-100 on the NPs dispersions, a solvent control was also performed, adding the same volume as used with the highest concentrations of NPs (0.2% of Triton X-100). The aqueous solutions of Triton X-100 or the NPs dispersions were added to the pre-moistened soil (in which water was added before), until 50% of the WHC maximum, and mixed manually [39]. The replicates were mixed individually as recommended by the OECD guideline [40]. Tests started 1 d after soil spiking. For the ERT extension, soil spiking was performed using the following nominal concentrations: 1, 10, 50, 100, and 500 mg BNPs or VNPs/kg soil. The concentrations that did not cause lethal effects (by ERT extension) were considered to the avoidance test. Therefore, soil spiking was performed using the following nominal concentrations: 10 and 50 mg BNPs/kg soil; 1 and 10 mg VNPs/kg soil.

### 2.4. Enchytraeid Reproduction Test Extension Procedures

The standard OECD guideline [39] was followed, with some adaptations (standard ERT extension) [41]. Briefly, 10 synchronized age organisms (17–19 d old) were introduced in each test container (Ø4 cm) with 20 g of moist soil and food supply (25 mg autoclaved oats). Tests ran over 28 d at 20 °C and a 16 h:8 h light:dark photoperiod. Food and water were replenished every week. Four replicates (n = 4) per experimental condition were used, plus one without organisms for abiotic factor measurement (e.g., pH). At the tests end, to extract organisms from soil and counting, replicates were fixated with 96% ethanol and Bengal rose (solution at 1% in ethanol). Samples were sieved through three meshes (0.6, 0.2, and 0.1 mm) to separate individuals from most of the soil and facilitate counting using a stereo microscope. Endpoints evaluated included survival and reproduction (number of adults and juveniles, respectively). Additionally, one replicate per condition was performed to monitor days 7, 14, and 21. For the 56 d exposure (ERT extension), four extra replicates were performed, and hence, larger test containers (Ø5.5 cm) were used with 40 g of soil per replicate because of the expected higher density of organisms. For these replicates, at day 28, adults were carefully removed from the soil, after which the soil was left, replenishing water and food weekly. At 56 d, the number of juveniles was assessed as performed for 28 d.

### 2.5. Avoidance Test Procedures

**Figure 1:**
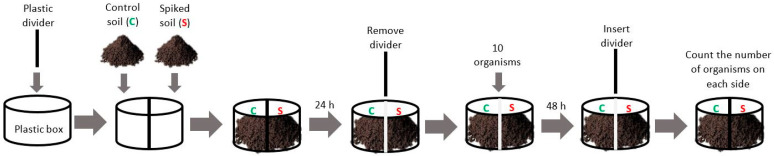
Schematic representation of the avoidance assay performed.

The avoidance test was performed following the earthworm avoidance test guideline [42] with some adaptations [43]. In short, containers (2.5 × 6.5Ø cm) with one removable plastic divider were used; each replicate contained 50 g of soil: one side with 25 g of control soil and the other side with 25 g of spiked soil. After this, the divider was gently removed (Figure 1). Test started 1 d after soil spiking when 10 adult organisms (with well-developed clitellum) were placed on the contact line of the soils. Boxes were covered with a plastic lid (containing small holes) and kept, for 48 h, at 20 °C with a photoperiod of 16 h:8 h (light:dark). Five replicates (n = 5) per experimental condition were used. An additional replicate per condition (without organisms) was prepared to measure the pH values (at the beginning and the end of the test). Five replicates with two sides containing control soil were also performed as a control approach to confirm the random distribution of the organisms. At the end of the test period, the divider was again inserted in the separation line between the two soils (control vs spiked) and each side of the box was independently searched for worms (Figure 1).

### 2.6. Data Analysis

Graphics and statistical analysis were performed using the Sigma Plot 12.5 software package. Shapiro-Wilk and Levene’s test were performed to assess the normality and homoscedasticity of data, respectively. To evaluate differences between control and NPs treatments, one-way analysis of variance (ANOVA), followed by Dunnett’s multiple comparison post hoc test, was applied. When data failed the normality and homoscedasticity tests, a non-parametric Kruskal-Wallis’ test was performed. Differences between control and solvent control were carried out using a Student *t*-test. Significant differences were accepted for a significance level (*p*) < 0.05. Toxicity Relationship Analysis Program (TRAP) 1.22 was used to fit data in adequate models and to calculate the Effect Concentrations (EC_x_).

The avoidance response expresses the percentage of affected worms (i.e., those which avoided the spiked soil) and was determined according to the earthworm avoidance test guideline [42]. Percentage of avoidance (%) per treatment was calculated as *A*:

$$A=\frac{C-T}{N}\times 100$$

where *C* is the number of organisms in the control soil; *T* is the number of organisms on the spiked soil; *N* is the total number of organisms used per replicate. Positive values indicate avoidance and negative values indicate a non-response or attraction to NPs. Percentages of avoidance (*A*) ≥80% indicate limited habitat function [42].

## 3. Results

### 3.1. Characterization of the Test Materials

**Figure 2:**
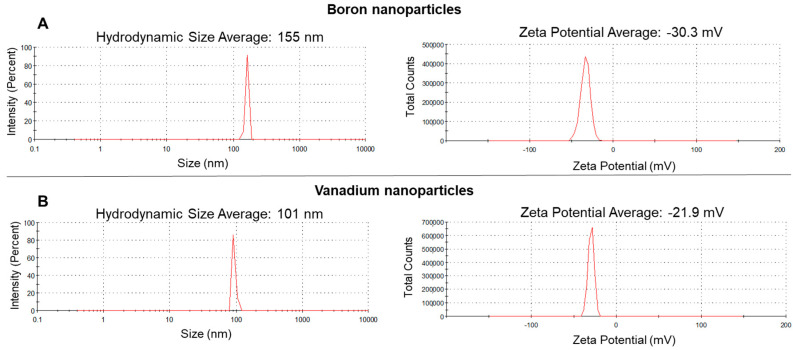
Hydrodynamic size assessed by dynamic light scattering and zeta potential evaluated by electrophoretic light scattering of boron nanoparticles (**A**) and vanadium nanoparticles (**B**) dispersions (at 0.02%, diluted in ultrapure water).

The characterization of NPs dispersions showed that the particles presented an average hydrodynamic size of 155 (PDI: 0.4) and 101 (PDI: 0.3) nm for BNPs and VNPs, respectively (Figure 2A,B). The ZP of both NPs was negative (Figure 2A,B).

### 3.2. Enchytraeid Reproduction Test Extension

For the ERT extension, there were no significant changes in soil pH within the test conditions or over the test duration (56 d). Moreover, there were no significant differences between the control and solvent control (*p* > 0.05). Therefore, the differences were assessed between treatments and the control group.

**Figure 3:**
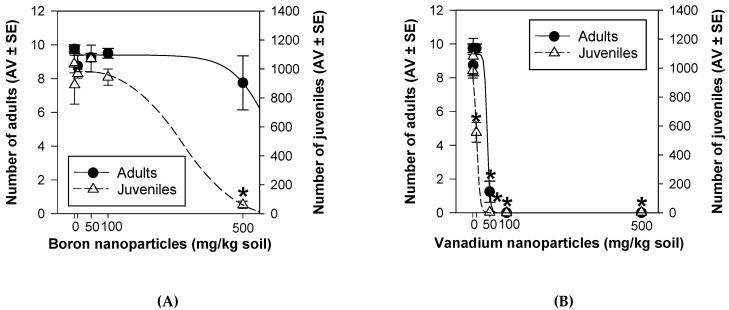
Effects of boron nanoparticles (**A**) and vanadium nanoparticles (**B**) spiked LUFA 2.2 soil on survival (number of adults) and reproduction (number of juveniles) of *Enchytraeus crypticus* after 28 days. Results are expressed as average value (AV) ± standard error (SE) (n = 4). Lines represent the models fit to data. * Significant differences to control (*p* < 0.05).

After 28 d, BNPs did not induce a significant effect on *E. crypticus* survival at the tested concentrations (*p* > 0.05; Figure 3A). However, BNPs, at 500 mg/kg, decreased the organisms’ reproduction (*p* < 0.05; Figure 3A). At 50, 100, and 500 mg/kg, VNPs decreased the *E. crypticus* survival and, consequently, their reproduction output was decreased (*p* < 0.05; Figure 3B). In addition, VNPs, at 10 mg/kg, decreased the organisms’ reproduction (*p* < 0.05; Figure 3B) but did not affect organisms’ survival (*p* > 0.05; Figure 3B).

**Figure 4:**
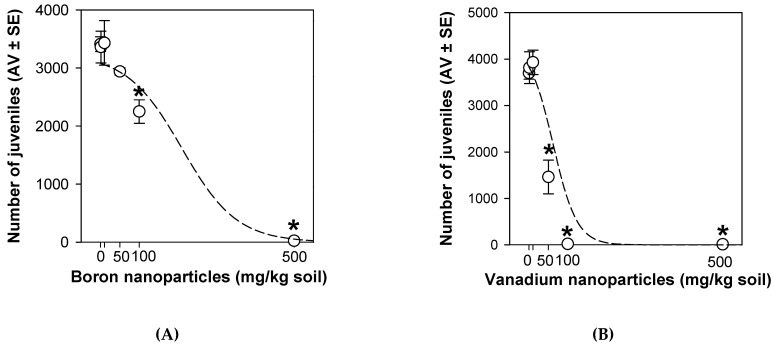
Effects of boron nanoparticles (**A**) and vanadium nanoparticles (**B**) spiked LUFA 2.2 soil on reproduction (number of juveniles) of *Enchytraeus crypticus* after 56 days. Results are expressed as average value (AV) ± standard error (SE) (n = 4). Lines represent the models fit to the data. * Significant differences to control (*p* < 0.05).

After 56 d, both NPs, at 100 and 500 mg/kg, decreased the *E. crypticus* reproduction (*p* < 0.05; Figure 4A,B). VNPs also decreased the reproduction at 50 mg/kg (*p* < 0.05; Figure 4B).

For survival, it was not possible to calculate a EC_x_ for BNPs due to the lack of effect detected in this endpoint (Table 2). In addition, for VNPs, no suitable model fit to the data from survival (Table 2). For reproduction, the calculated EC_x_ values were higher for BNPs than VNPs at both exposure times (28 and 56 d) (Table 2).

**Figure 5:**
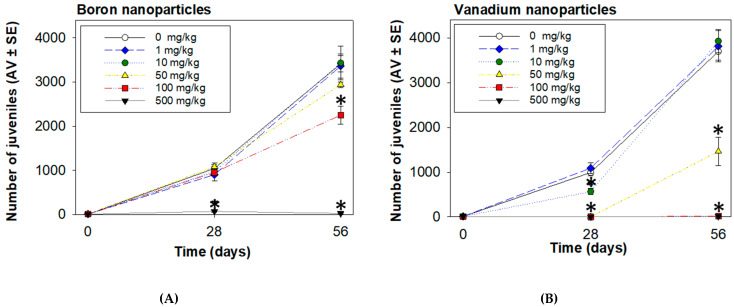
Effects on *Enchytraeus crypticus* reproduction, after 28 vs. 56 d exposure to boron nanoparticles (**A**) and vanadium nanoparticles (**B**) in LUFA 2.2 soil. Results are expressed as average value (AV) ± standard error (SE) (n = 4). * Significant differences to control (*p* < 0.05).

The calculated BNPs EC_x_ for reproduction were lower at 56 d comparing with 28 d, whereas, for VNPs, an opposite pattern was found: EC_x_ at 56 d were higher than ECx at 28 d (Table 2). With the extension of the exposure time, for BNPs, effects on the reproduction occurred at lower concentrations at 56 d than at 28 d (Figure 5A). Specifically, 100 mg/kg of BNPs did not decrease the organisms’ reproduction at 28 d but decreased at 56 d (Figure 5A). For VNPs, the decrease on the organisms’ reproduction occurred at low concentrations at 28 d compared with 56 d (Figure 5B). Namely, 10 mg/kg of VNPs induced a negative effect in this endpoint at 28 d, whereas this effect disappeared at 56 d (Figure 5B).

### 3.3. Avoidance Test

There were no significant changes in soil pH within the test conditions or over the test duration (48 h). Moreover, there were no significant differences between the control and solvent control (*p* > 0.05). Therefore, the differences were assessed between treatments and the control group.

**Figure 6:**
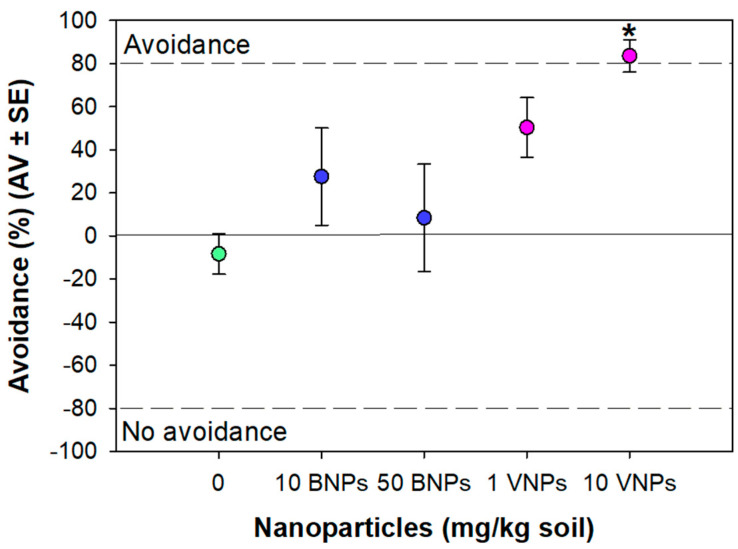
Avoidance responses of *Enchytraeus crypticus* after 48 h exposure to boron nanoparticles (BNPs) and vanadium nanoparticles (VNPs) in LUFA 2.2 soil. Results are expressed as average value (AV) ± standard error (SE) (n = 5). * Significant differences to control (*p* < 0.05).

Considering the tested conditions, there was a tendency of the organisms to avoid the VNPs-contaminated soils, with this response being significantly different from the control at 10 mg/kg (83.6% of avoidance—Figure 6). BNPs did not significantly induce avoidance responses of the organisms compared with control group (*p* > 0.05; Figure 6).

## 4. Discussion

Understanding the physicochemical characteristics of NPs in the receiving medium is particularly important since they affect NPs’ fate, behavior, and consequently their toxicity [44]. However, in the present study, the characteristics of both NPs were assessed in ultrapure water, because this assessment in environmental matrices, such as soil, is a challenge due to a lack of adequate and reliable protocols [23,44,45]. The hydrodynamic size in ultrapure water were 155 and 101 nm for BNPs and VNPs, respectively. The hydrodynamic size of BNPs was relatively higher than the APS provided by the supplier (between 80 and 100 nm), which may suggest that, in the colloidal suspensions, BNPs became clustered into larger structures. The ZP values of both NPs in ultrapure water were negative (−30.3 and −21.9 mV, for BNPs and VNPs, respectively), indicating that the surfaces of the NPs were negatively charged and, in general, close or lower than −30 mV, which may indicate colloidal stability. NPs strong positive or negative ZP values (in general, >30 mV or <−30 mV) indicate good physical stability of nanosuspensions due to electrostatic repulsion of individual particles. In general, the ZP magnitude reveals the colloidal system’s potential stability: if all the particles in suspension have a strong negative or positive ZP, they will repel one another and have no tendency to come together [46]. However, the presence of the nonionic surfactant Triton X-100 in the NPs dispersions must be taken into consideration because it can influence the ZP of NPs. In general, as previously described, the presence of nonionic surfactants, as stabilizers in NPs dispersions, decreases the absolute magnitude of ZP [47].

In terms of lethality, after 28 d, BNPs did not affect *E. crypticus* survival. A study with BNPs using bees (*A. melifera*) reported LC_50_ values of 229.1 and 0.339 mg/L for 48 and 96 h exposure, respectively, showing higher lethality with the increase of time [12]. Concerning aquatic organisms, BNPs induced 100% mortality in *D. magna* for concentrations above 80 mg/L, after 24 h [13]. A BNPs LC_50_ for *Vibrio fischeri* ranging from 56 to 66 mg/L, depending upon aging time/age of solution, was calculated [13]. Therefore, BNPs can be considered as ‘‘harmful’’ to aquatic microorganisms according to the Commission Directive 93/67/EEC from the European Union. These distinct results in terms of lethality, compared with our study, may be due to the used distinct species, exposure routes, and NPs characteristics. On the other hand, after 28 d, VNPs decreased *E. crypticus* survival at 50, 100, and 500 mg/kg. No previous in vivo study was found about the effects of VNPs. Only an in vitro research showed a higher decrease in A549 cell viability (97%) at concentrations of 100 μg V trioxide (V_2_O_3_) NPs/mL [16].

Regarding reproduction, BNPs affected this endpoint after 28 d at 500 mg/kg and after 56 d at 100 and 500 mg/kg. Although in our work B in nano form was used, it was previously reported that B particulates (i.e., not nanosized particles), at lower levels, positively contributed to diverse physiological effects on vertebrates (embryogenesis, immunity, and psychomotor functions) [48,49], but it was toxic at higher levels [50]. Studies showed that B particulates at 12.5 and 25 mg/day decreased the testosterone levels in rats [51,52], while lower doses did not disturb. Additionally, a two-generational study found seminiferous tubule degeneration and spermatogenesis impairment in the CD-1 mice when the progenitors were fed with ≥111 mg B/kg/day [53]. These data showed that B particulates may have negative implications in the reproduction performance, as it was observed in the present study, where a decrease in the *E. crypticus* fertility was found. VNPs also affected the *E. crypticus* reproductive output after 28 d (at 10, 50, 100, and 500 mg/kg) and 56 d (at 50, 100, and 500 mg/kg). In fact, it is well established that vanadate (V^5+^) and vanadyl (V^4+^) may affect reproduction and development in mammals and decrease fertility, inducing embryolethality, fetotoxicity, and teratogenicity in rats, mice, and hamsters [54]. For both NPs, reproduction was more disturbed than survival, indicating the reproductive output as the most sensitive. This finding was already found for *E. crypticus* testing the effects of tungsten carbide cobalt NPs (WCCoNPs) [32], silver NPs (Ag NM300K) [29], and nickel NPs (NiNPs) [34]. Specifically, NiNPs caused delayed maturation and decreased the growth of *E. crypticus* [34], which may explain the reduction in the organisms’ reproduction. 

Comparing the toxicity of BNPs with VNPs, the latter caused, in general, more adverse effects, showing that the mechanisms of toxicity are dependent on the nature of NPs. In terms of survival, BNPs did not cause a significant effect, whereas VNPs caused organisms mortality. In terms of reproduction: EC_x_ for BNPs > EC_x_ for VNPs. The results revealed that the toxicity of the tested NPs can be determined by their physico-chemical properties and mechanisms, e.g., particle nature (metalloid (BNPs) versus metal (VNPs)-based NPs). Early studies already showed distinct effects on *E. crypticus* considering different natures of NPs, e.g., after 28 d, WCCoNPs caused no effect in survival and a decrease in reproduction at 1600 mg/kg [32]; after 21 d, Ag NM300K decreased survival at concentrations ≥600 mg/kg and reproduction at concentrations ≥200 mg/kg [29]; after 21 d, NiNPs induced no effect in survival but decreased reproduction at concentrations ≥700 mg/kg [34]. With the increase of NPs concentration, the toxicity of BNPs and VNPs, in general, also increased for all the evaluated endpoints: survival, reproduction, and avoidance. A similar pattern was already found in previous studies, specifically, ERT with Ag NM300K [29], ERT and full life cycle (FLC) with NiNPs [34].

The toxicity of BNPs and VNPs (metalloid and metal-based NPs, respectively) may be explained by the toxicity of the NPs themselves and/or by the products resultant from the dissolution of the NPs. The trace mineral B is inert to air and water at room temperature; thus, being insoluble in water [55], it will not be easily ionized, and therefore the effects observed on the reproduction by the BNPs exposure must be linked to the presence of nanoparticulates per se (e.g., incorporated by the organisms and interacting with biomolecules). However, regarding VNPs, a study reported that V oxide (VO_2_) and V pentoxide (V_2_O_5_) NPs showed higher dissolution rates determining toxicity by imbalance of ion homeostasis in the organism [56]. Therefore, as described for other metal NPs (such as AgNPs [57]), the toxicity induced by VNPs may be due to the release of ions (in this case V ions) and not due to the toxicity of the NPs themselves. In the present study, the tested VNPs were not at an oxidation state. However, V is a transitional state element that may be converted in four oxidation states: V^2+^, V^3+^, VO^2+^, and VO_4_^2−^. Hence, the VNPs toxicity can be determined by the oxidation state [16] and the tendency (or not) for oxidation status can affect the NPs toxicity. A study using a lung cell line A549 reported that dissolution of VO_2_NPs played a key role in the cytotoxicity [14]. However, in the present study, the detected effects induced by VNPs may have also been caused by a nanoparticulate-specific effect (as described for other metal-based NPs [58]), possibly because: (1) VNPs may damage the membrane of cocoons or the epithelium of the juveniles/adults with consequent embryos/organisms mortality; (2) VNPs may cross the membrane of cocoons and organisms, damaging the tissues by a release of V ions [29].

Long-term exposures are considered highly relevant scenarios for persistent materials such as NPs. The present study showed that the mechanisms of toxicity of the tested NPs are dependent on the exposure duration. In general, BNPs and VNPs showed different effects at 28 d from the ones detected at 56 d, dependent on the type of NPs (B versus V). The effects of BNPs became worse through the time of exposure (for reproduction: EC_x_ 56 d < EC_x_ 28 d) whereas effects of VNPs were alleviated along the time of exposure (for reproduction: EC_x_ 56 d > EC_x_ 28 d). In fact, increasing the exposure period to 56 d (i.e., duplicating the time to include a second generation), a reduction in the descendants number was also found at the second higher-tested BNPs concentration (100 mg/kg soil), which suggests a toxicological mechanism associated with the progenitors, i.e., a parental effect, as also mentioned in studies with NPs of WCCo, Ag, and Ni [29,32,34]. Nevertheless, using lung cells, a long-term exposure (20 d) showed that the VO_2_NPs were harmful at lower doses when compared with a short-term exposure period (1 d) [14]. This result may indicate a temporal alteration of VO_2_NPs, which culminates in adverse effects, induced by lower doses, as opposed to our results. At 56 d, an enhancement in the organisms’ reproduction was seen for 10 mg VNPs/kg, a concentration that negatively affected the reproduction at 28 d. Thus, it seems that, increasing the exposure period, organisms exposed to 10 mg VNPs/kg were able to efficiently activate the antioxidant defense and/or repair mechanisms, allowing them to detoxify and hatch. For the highest concentrations, 100 and 500 mg VNPs/kg, the level of antioxidant protection and/or repair systems was not enough, since the reproduction decrease was detected at 28 and 56 d. The role of V in oxidative stress was recently reviewed [59].

Various studies have shown that long-term exposures (in specific multigenerational exposures, FLC test, and ERT standard extension) allow for the discrimination of effects not predictable in short-term studies [28,34,36,41]. In particular, a FLC test with copper oxide (CuO) NPs showed an increased organisms’ sensitivity, e.g., reproductive effects, compared with the standard ERT (FLC test: EC_10_ = 8 mg/kg; standard ERT: EC_10_ = 421 mg/kg). This is in line with BNPs effect, i.e., 100 mg BNPs/kg did not cause a significant effect on the organisms’ reproduction at 28 d but decreased the reproduction at 56 d, showing that the mechanisms of defense were not enough to protect the organisms over time, at concentrations ≥100 mg/kg.

VNPs, at 10 mg/kg, induced avoidance behavior, as opposed to BNPs. Once again, VNPs caused more effects on *E. crypticus* than BNPs, supporting the results found from ERT standard extension. The avoidance percentage detected at 10 mg VNPs/kg was >80%, representing limited habitat function on the VNPs-spiked soils. Some studies already reported the potential of other NPs (in specific: titanium silicon oxide [20], Ag [26], cerium dioxide [22], and silica [23]) to induce avoidance behavior in soil organisms. However, the avoidance responses were found at higher concentrations of NPs (≥36 mg/kg) [20,22,23,26] than the one found in the present study (10 mg/kg). This result shows the high sensitivity of the organisms’ chemoreceptors to detect the presence of VNPs-contaminated soil and consequently trigger an avoidance response. For the avoidance to occur, the danger must be first perceived, which may not happen if the organism is ‘blinded’ in some capacity. The danger may be not recognized if the organisms’ chemoreceptors are impaired [23,60]. The absence of BNPs effect on the behavior of *E. crypticus* agrees with an earlier study assessing the effects of boric acid [43]. Bicho et al. (2015) [41] associated the non-avoidance behavior of *E. crypticus* with the gamma-aminobutyric acid (GABA) system. The up-regulation of the GABA is known to trigger anaesthetic effects [43]; hence, this may at least partially explain the obtained results from the present study. A correlation between non-avoidance and acetylcholinesterase inhibition was also previously reported for soil organisms [21].

In the current study, the measured endpoints were restricted to survival, reproduction, and avoidance-adverse outcomes; hence, a refined understanding was limited. However, data from the assessed parameters will greatly contribute to the evaluation of NPs risk assessment. Further studies are encouraged, specifically: (1) performing a FLC test to discriminate the effects between life stages (cocoons, juveniles, and adults), including endpoints, such as hatching, maturity status, and growth, besides population estimates like instantaneous growth rate among others; (2) longer-term exposures assessing the correspondent water-soluble compounds (e.g., boric acid and V salts) to compare effects of nano versus non-nano forms; (3) assessment of endpoints at molecular and biochemical levels to clarify the observed phenotypic effects (e.g., reproduction reduction and avoidance behavior).

## 5. Conclusions

VNPs caused, in general, more adverse effects than BNPs, showing that the mechanisms of toxicity are dependent on the nature of NPs. In terms of survival, BNPs did not cause significant effects, whereas VNPs caused mortality (≥50 mg/kg). In terms of reproduction, the calculated EC_x_ for BNPs > EC_x_ for VNPs. Specifically, the 28 and 56 d EC_50_ values were 319 and 210 mg/kg for BNPs and 11 and 62 mg/kg for VNPs. The present study also showed that the mechanisms of toxicity of the tested NPs are dependent on the exposure period, showing the relevance in the implementation of long-term studies. The BNPs toxic effects increased with the time of exposure (from 28 to 56 d), whereas VNPs effects were alleviated. VNPs, at 10 mg/kg, induced avoidance behavior, > 80%, representing limited habitat function, contrarily to BNPs (no effect). Obtained data can contribute to the improvement of NPs risk assessment, because it was possible to assess the toxic effects of BNPs and VNPs on *E. crypticus*.

## Figures and Tables

**Table 1:** Studies assessing the long-term effects of engineered nanoparticles to the terrestrial species *Enchytraeus crypticus*. Rf—Reference; EC_10_ and EC_50_—Concentration that causes 10 and 50% of the effect, respectively.

Exposure Characteristics	Assessed Endpoints	Main Findings	Rf
Copper Oxide Nanoparticles (CuONPs)			
Multigenerational (MG) exposure 1 year	- Survival - Reproduction	- CuONPs increased toxicity for EC_10_ exposed organisms; - CuONPs showed mechanisms of toxicity in the longer-term exposures, not predictable based on short-term studies.	[28]
Full life cycle (FLC) test 46 days (d)	- Hatching - Growth - Maturity - Survival - Reproduction	- CuONPs caused toxicity during the juvenile stage, reducing growth, maturation, and reproductive output; - EC_50_ maturity status (25 d): 3833 mg/kg; - EC_50_ reproduction (46 d): 1075 mg/kg.	[36]
MG exposure 224 d	- Global DNA methylation - Gene-specific methylation - Gene expression	- CuONPs increased global DNA methylation; - Changes in the epigenetic, stress, and detoxification gene targets, also occurring in post-exposure generations.	[37]
FLC test + MG exposure 46 and 224 d	- Histology - Immuno-histochemistry	- No tissue alterations; - CuONPs affected the Notch signaling pathway.	[31]
Lifespan test 202 d	- Survival - Reproduction	- CuONPs caused shorter life of the adults; - A more amplified effect was found in terms of reproduction.	[35]
Nickel Nanoparticles (NiNPs)			
FLC test 46 d	- Hatching - Growth - Maturity - Survival - Reproduction	- Hatching was the most sensitive endpoint, although the organisms recovered; - EC_50_ hatching (11 d): 870 mg/kg; EC_50_ growth (25 d): > 3200 mg/kg; EC_50_ maturity status (25 d): 3946 mg/kg; EC_50_ survival (46 d): 3627 mg/kg; EC_50_ reproduction (46 d): 3455 mg/kg.	[34]
Silver Nanoparticles (Ag NM300K)			
FLC test 46 d	- Hatching - Growth - Maturity - Survival - Reproduction	- Ag NM300K caused a non-monotonic concentration-response effect; - EC_50_ hatching (11 d): 61 mg/kg; EC_50_ maturity status (25 d): 131 mg/kg; EC_50_ survival (46 d): 99 mg/kg; EC_50_ reproduction (46 d): 103 mg/kg.	[29]
Tungsten Carbide Cobalt Nanoparticles (WCCoNPs)			
Enchytraeid Reproduction Test extension 56 d	- Survival - Reproduction	- WCCoNPs caused no effect on survival; - EC_50_ reproduction (28 d): 1500 mg/kg; EC_50_ reproduction (56 d): 128 mg/kg.	[32]
MG exposure 224 d	- Survival - Reproduction	- MG exposure did not increase toxicity; - An increase in reproduction at low concentrations of WCCoNPs was found.	[33]
MG exposure 224 d	- Global DNA methylation	- MG exposure increased global DNA methylation, which continued in unexposed generations and was associated with an increase in reproduction.	[30]

**Table 2:** Effect Concentrations (EC_x_), applying the 2-parameters Logistic model, for *Enchytraeus crypticus* survival, assessed at 28 days, and reproduction, assessed at 28 and 56 days, after exposure to boron nanoparticles (BNPs) and vanadium nanoparticles (VNPs) in LUFA 2.2 soil. EC_20_, _50_ and _80_: Concentration that causes 20%, 50%, and 80% of the effect, respectively. n.e.—no effect; n.d.—not possible to determine. Results are presented as estimated value ± standard error.

Test Materials	EC_20_ (mg/kg)	EC_50_ (mg/kg)	EC_80_ (mg/kg)
Survival at 28 d			
BNPs	n.e.	n.e.	n.e.
VNPs	n.d.	n.d.	n.d.
Reproduction			
BNPs			
28 d	217.0 ± 79.4	319.0 ± 59.5	393.8 ± 72.8
56 d	111 ± 32.4	210.0 ± 70.8	308.0 ± 115.1
VNPs			
28 d	5.0 ± 1.4	11.0 ± 1.5	18.0 ± 3.0
56 d	19.0 ± 9.8	62.0 ± 9.0	105.0 ± 15.8

## Data Availability

The data are available on request from the corresponding author.
